# Feasibility of PET/CT system performance harmonisation for quantitative multicentre ^89^Zr studies

**DOI:** 10.1186/s40658-018-0226-7

**Published:** 2018-11-21

**Authors:** Andres Kaalep, Marc Huisman, Terez Sera, Danielle Vugts, Ronald Boellaard, on behalf of EARL, EATRIS, the TRISTAN Consortium (#IB4SD-116106)

**Affiliations:** 1 Department of Medical Technology, North Estonia Medical Centre Foundation, J. Sutiste Str 19, 13419 Tallinn, Estonia; 2 Department of Radiology and Nuclear Medicine, VU University Medical Center, Amsterdam, The Netherlands; 3 Department of Nuclear Medicine, University of Szeged, Szeged, Hungary; 4 Department of Nuclear Medicine and Molecular Imaging, University of Groningen, University Medical Center Groningen, Hanzeplein 1, Groningen, The Netherlands; 5 EANM Research Limited (EARL), Vienna, Austria; 6 EATRIS ERIC (European Infrastructure for Translational Medicine), Amsterdam, The Netherlands; 7 TRISTAN-IMI consortium (Translational Imaging in Drug Safety Assessment - Innovative Medicines Initiative), Amsterdam, The Netherlands

**Keywords:** ^89^Zr, Performance, Harmonisation, PET/CT, Quantification, EARL accreditation

## Abstract

**Purpose:**

The aim of this study was to investigate the variability in quantitative performance and feasibility of quantitative harmonisation in ^89^Zr PET/CT imaging.

**Methods:**

Eight EANM EARL-accredited (Kaalep A et al., Eur J Nucl Med Mol Imaging 45:412–22, 2018) PET/CT systems were investigated using phantom acquisitions of uniform and NEMA NU2-2007 body phantoms. The phantoms were filled according to EANM EARL guidelines for [^18^F]FDG, but [^18^F]FDG solution was replaced by a ^89^Zr calibration mixture. For each system, standard uptake value (SUV) accuracy and recovery coefficients (RC) using SUVmean, SUVmax and SUVpeak metrics were determined.

**Results:**

All eight investigated systems demonstrated similarly shaped RC curves, and five of them exhibited closely aligning recoveries when SUV bias correction was applied. From the evaluated metrics, SUVpeak was found to be least sensitive to noise and reconstruction differences among different systems.

**Conclusions:**

Harmonisation of PET/CT scanners for quantitative ^89^Zr studies is feasible when proper scanner-dose calibrator cross-calibration and harmonised image reconstruction procedures are followed. An accreditation programme for PET/CT scanners would facilitate multicentre ^89^Zr quantitative studies.

## Introduction

The use of radiolabelled antibodies for diagnostic and therapeutic purposes has been going on for more than 50 years [1]. Their application as imaging probes in positron emission tomography (PET) combines the high sensitivity of PET with the high antigen specificity of monoclonal antibodies [2]. ^89^Zr-based tracers are becoming widespread with increasingly available supply, advances in radiochemistry and successful pilot studies in humans. However, multicentre studies using ^18^F-labelled tracers have demonstrated the need for standardisation of image acquisition, reconstruction, and analysis procedures and international harmonisation programmes such as EANM and EARL aim to facilitate the use of FDG PET as a quantitative imaging biomarker [3, 4]. A detailed discussion on ^89^Zr physics in PET has been published by Conti et al. [5].

The aim of this study was to investigate the variability in quantitative performance and feasibility of quantitative harmonisation in ^89^Zr PET/CT imaging.

## Materials and methods

### Investigated systems and phantom experiments

Eight PET/CT systems (system 1–8), calibrated according to the manufacturer’s instructions, while also participating and accredited in the EANM/EARL [^18^F]FDG PET/CT accreditation programme, were selected for this study. The investigated systems were two General Electric Discovery 690, two General Electric Discovery 710, one Siemens Biograph 40 mCT, one Siemens Biograph 64 mCT, one Siemens Somatom Definition AS mCT and one Philips Ingenuity TF.

**Fig. 1:**
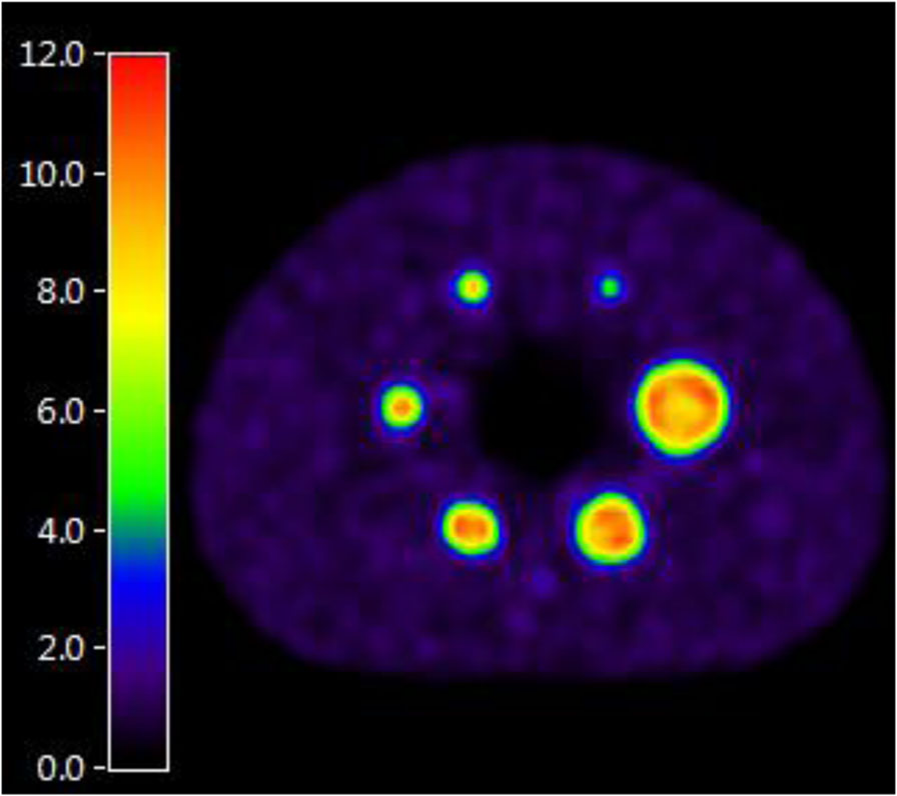
Transversal slice of ^89^Zr-filled NEMA NU2-2007 body phantom

Two phantom experiments were carried out in accordance with EANM/EARL guidelines—Calibration QC and NEMA Phantom QC—where [^18^F]FDG was substituted with a ^89^Zr calibration sample. In the first experiment, a uniform cylindrical phantom was filled with a solution containing 8–12 kBq/mL of ^89^Zr. In the second experiment, the NEMA NU2-2007 body phantom background compartment and spheres were filled with a ^89^Zr solution of 2 kBq/mL and 20 kBq/mL, respectively, so as a 10:1 sphere to background ratio can be achieved (Fig. 1). Exact amount of ^89^Zr activity was measured for each scan using only local dose calibrators, which had not underwent specific cross-calibration for ^89^Zr. In both experiments, the phantoms underwent a low-dose CT acquisition followed by PET acquisition of two consecutive bed positions of 5 min each. Images were reconstructed using EARL-compliant parameters routinely used by the corresponding sites for [^18^F]FDG quantitative imaging (Table 1).

**Table 1:** Reconstruction settings

System	System	Pixel spacing (mm)	Slice thickness (mm)	Reconstruction method	Post filter width (mm)	Resolution recovery
A	Discovery 690	3.65	3.27	VPFXS	6.4	Yes
B	Discovery 690	2.73	3.27	VPHD	6.5	No
C	Discovery 710	2.73	3.27	VPFX	6.4	No
D	Discovery 710	2.73	3.27	VPFXS	9.0	Yes
E	Biograph 40 mCT	3.18	2.00	PSF+TOF	7.0	Yes
F	Somatom Definition AS mCT	3.18	5.00	PSF+TOF	8.0	Yes
G	Ingenuity TF PET/CT	4.00	4.00	BLOB-OS-TF	4.0	No
H	Biograph 64 mCT	4.07	5.00	OSEM3D+TOF	5.0	No

### Data analysis

Reconstructed DICOM images were analysed using the EARL semi-automatic tool [3, 6] designed for quantitative analysis of images of uniform and NEMA NU2-2007 body phantoms. From the uniform phantom and the NEMA body phantom’s uniform background compartment, SUV accuracies for each system were determined. From the NEMA body phantom experiments, recovery coefficients (RC) were calculated as a function of sphere sizes, defined as ratio of activity concentration estimated from PET images to the expected activity concentration measured by dose calibrator. Different RC metric values were calculated based on 50% background-corrected isocontour VOI (SUVmean), maximum voxel value included in VOI (SUVmax) and spherical VOI with a diameter of 12 mm, positioned so as to yield the highest uptake (SUVpeak) [6–8]. Using data from the EARL database, relevant FDG RC curves of the corresponding scanners are displayed as a reference.

Additionally, RC curves were rescaled to correct for a global SUV bias, derived from the phantom’s background compartment, to mitigate the impact of cross-calibration error between PET/CT system and dose calibrator on the observed RC. In order to directly compare the RC curves’ shapes of all systems, the individual recovery coefficients of the NEMA body phantom spheres were normalised to the recovery coefficient of the largest (37 mm) sphere.

## Results

**Fig. 2:**
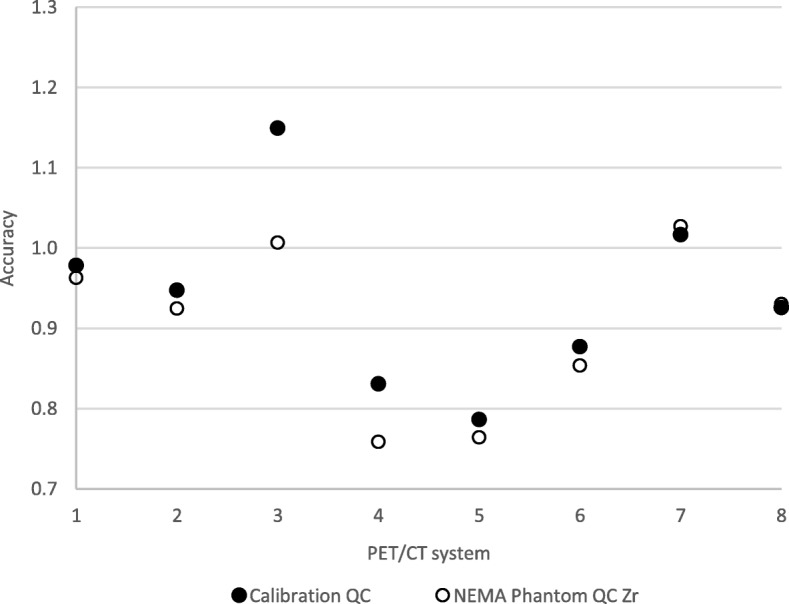
SUV accuracy of the PET scanners relative to the dose calibrator measurements plotted by system number. Systems 1–2 and 3–4 represent GE scanners Discovery 690 and Discovery 710, respectively; systems 5–6 are Siemens Biograph 40_mCT and Somatom Definition AS_mCT; system 7 is the Philips Ingenuity TF PET/CT; system 8 is the Siemens Biograph 64_mCT

**Fig. 3:**
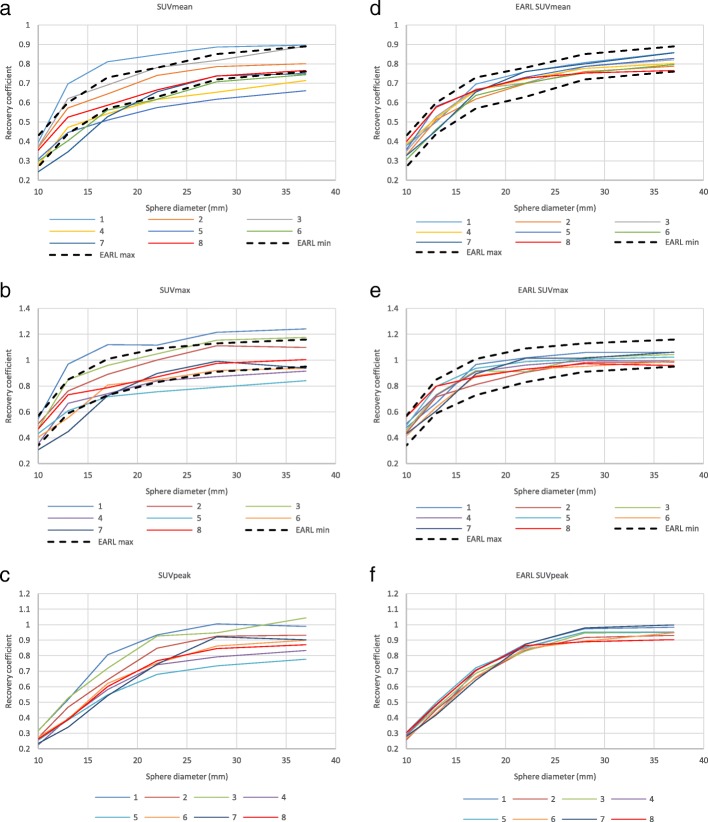
RC curves derived from the ^89^Zr phantom experiments using SUVmean (**a**), SUVmax (**b**) and SUVpeak (**c**) quantitative metrics and corresponding RC curves derived from the EARL [^18^F]FDG phantom experiments using SUVmean (**d**), SUVmax (**e**) and SUVpeak (**f**) quantitative metrics. Current EARL specifications for [^18^F]FDG-PET/CT accreditation are presented as bold dashed lines. Systems 1–2 and 3–4 represent GE scanners Discovery 690 and Discovery 710, respectively; systems 5–6 are Siemens Biograph 40_mCT and Somatom Definition AS_mCT; system 7 is the Philips Ingenuity TF PET/CT; system 8 is the Siemens Biograph 64_mCT

**Fig. 4:**
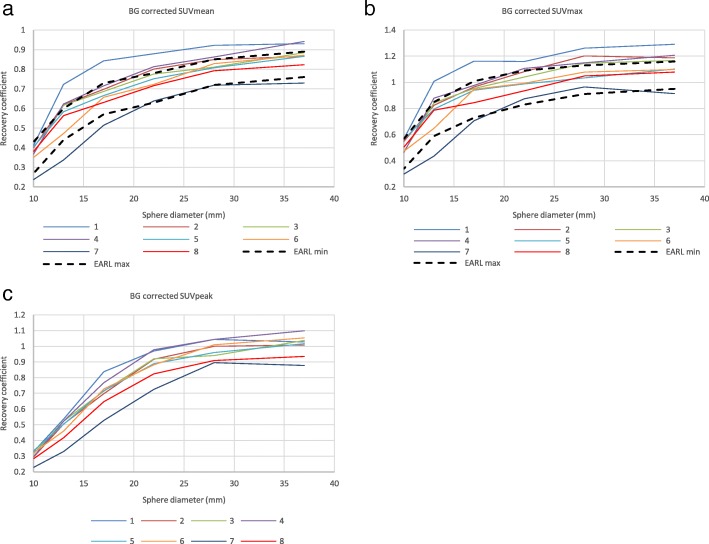
Background SUV bias-corrected RC curves derived from the phantom experiments using SUVmean (**a**), SUVmax (**b**) and SUVpeak (**c**) quantitative metrics. Current EARL specifications for [^18^F]FDG-PET/CT accreditation are presented as bold dashed lines. Systems 1–2 and 3–4 represent GE scanners Discovery 690 and Discovery 710, respectively; systems 5–6 are Siemens Biograph 40_mCT and Somatom Definition AS_mCT; system 7 is the Philips Ingenuity TF PET/CT; system 8 is the Siemens Biograph 64_mCT

**Fig. 5:**
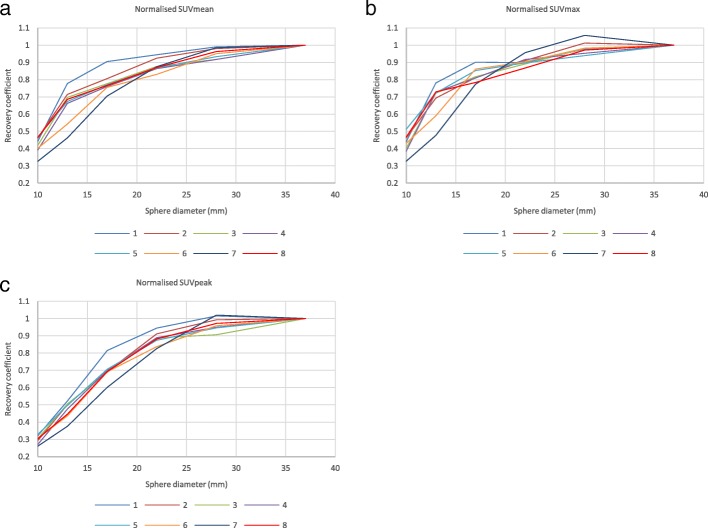
RC curves normalised to the largest sphere, derived from the phantom experiments using SUVmean (**a**), SUVmax (**b**) and SUVpeak (**c**) quantitative metrics. Systems 1–2 and 3–4 represent GE scanners Discovery 690 and Discovery 710, respectively; systems 5–6 are Siemens Biograph 40_mCT and Somatom Definition AS_mCT; system 7 is the Philips Ingenuity TF PET/CT; system 8 is the Siemens Biograph 64_mCT

The SUV bias from both phantom experiments is presented in Fig. 2. The results for SUVmean, SUVmax and SUVpeak together with corresponding information for EARL [^18^F]FDG can be seen in Fig. 3, while the results corrected for SUV bias calculated from the body phantom background are presented in Fig. 4. Figure 5 demonstrates the RC curves normalised to the largest 37-mm sphere recovery.

## Discussion

In order to remain in the optimal measurement range of the dose calibrators, the ^89^Zr activity used in the study was similar to what is injected to a patient in clinical practice, resulting in significantly higher activity concentrations in the phantoms compared to patients (due to the smaller phantom volumes). However, lower counts are expected to further increase the variability of the results and may have hampered comparing recoveries between systems, with current EARL specifications and with those seen with ^18^F. For clinical studies, low count rates potentially induce an upward bias when SUVmax is used. To mitigate this upward bias, SUVpeak is an alternative, which is less sensitive to scanner variation and image noise, and might therefore be the optimal metric to assess tracer uptake for ^89^Zr. Consequently, in our phantom study, we included SUVpeak as well.

In addition to verifying the results of a recent study by Makris et al. [9], current study investigated the real-life scenario of using only local dose calibrators for ^89^Zr measurement as well as by asking sites to perform the experiments themselves using the provided manuals and instructions. Out of the eight systems investigated in total, four Calibration QC and three NEMA Phantom QC experiments demonstrate a SUV bias of > 10% (Fig. 2). Since the scanners are EARL accredited for [^18^F]FDG-PET/CT, they comply with accreditation specifications for SUV bias (≤ 10%); it is therefore believed that the large global errors are due to inaccurate cross-calibration between the scanners and dose calibrators used to measure the ^89^Zr solution activity on site. While each of the dose calibrators should be set up by the manufacturer to accurately measure ^89^Zr, the results from our study underline the importance of a traceable calibration performance of dose calibrators used in ^89^Zr quantitative PET/CT imaging.

From Fig. 2, it can be seen that SUV bias values derived from Calibration QC and NEMA Phantom QC background agree reasonably well, with the exception of only system 3 and to some extent system 4. These inconsistencies as well as the variable bias in RC curves (Fig. 3) are suggested to be related to activity measurement and phantom filling procedures on site.

The initial RC curves derived from the images (Fig. 3, a–c) demonstrate increased spread compared to the background-corrected ones (Fig. 4). After applying the SUV bias correction, the RC values of five systems show good alignment with each other and with EANM specifications for [^18^F]FDG. Two of the investigated systems (1 and 7) remain out of specifications even after correcting for SUV bias. The reason for this is unknown and would need further investigation. RC curves normalised to the largest (37 mm) sphere (Fig. 5) demonstrate similar shapes of RC curves for all investigated systems. This would suggest that, with further adjustment—meaning reduction of global SUV bias based on Calibration QC experiment data and possibly minor adjustment of the image reconstruction parameters—all of the systems should be able to achieve harmonisation.

The closest alignment of the RC curves can be observed when SUVpeak is used. This demonstrates the potential of this metric being used when quantitative harmonisation is desired. It should however be noted that with the use of SUVpeak, one should expect a decrease in overall contrast recovery, compared to SUVmax.

Finally, with the shape of ^89^Zr RC curves shown to be similar to ^18^F in our pilot study, further harmonisation efforts could be focused on the cross-calibration of the dose calibrators, which is considered to be the largest source of uncertainty in this case. A future ^89^Zr harmonisation scheme could therefore be based on a ^89^Zr dose calibrator cross-calibration quality control, with a successful site [18F]FDG EARL accreditation being a prerequisite.

## Conclusions

All eight investigated systems demonstrated similarly shaped RC curves, and five of them exhibited close alignment when SUV bias correction was applied. Use of SUVpeak as a metric, which proved to be the least sensitive to noise and reconstruction differences among systems, is strongly recommended for multicentre quantitative ^89^Zr studies. When PET/CT and dose calibrator cross-calibration procedures are closely followed and the image reconstruction parameters adjusted, the quantitative harmonisation of scanners for ^89^Zr PET studies is feasible. Yet, our results demonstrate the urgent need to set up a suitable cross-calibration and accreditation programme to facilitate multicentre ^89^Zr quantitative studies.
